# Functional Roles of CD133: More than Stemness Associated Factor Regulated by the Microenvironment

**DOI:** 10.1007/s12015-023-10647-6

**Published:** 2023-11-03

**Authors:** Angela Patricia Moreno-Londoño, Martha Robles-Flores

**Affiliations:** Department of Biochemistry, Facultad de Medicina, Universidad Nacional Autónoma de México (UNAM), 04510 Mexico City, Mexico

**Keywords:** Cancer stem cell, Stemness associated markers, CD133, Tumor microenvironment

## Abstract

CD133 protein has been one of the most used surface markers to select and identify cancer cells with stem-like features. However, its expression is not restricted to tumoral cells; it is also expressed in differentiated cells and stem/progenitor cells in various normal tissues. CD133 participates in several cellular processes, in part orchestrating signal transduction of essential pathways that frequently are dysregulated in cancer, such as PI3K/Akt signaling and the Wnt/β-catenin pathway. CD133 expression correlates with enhanced cell self-renewal, migration, invasion, and survival under stress conditions in cancer. Aside from the intrinsic cell mechanisms that regulate CD133 expression in each cellular type, extrinsic factors from the surrounding niche can also impact CD33 levels. The enhanced CD133 expression in cells can confer adaptive advantages by amplifying the activation of a specific signaling pathway in a context-dependent manner. In this review, we do not only describe the CD133 physiological functions known so far, but importantly, we analyze how the microenvironment changes impact the regulation of CD133 functions emphasizing its value as a marker of cell adaptability beyond a cancer-stem cell marker.

**Graphical Abstract:**

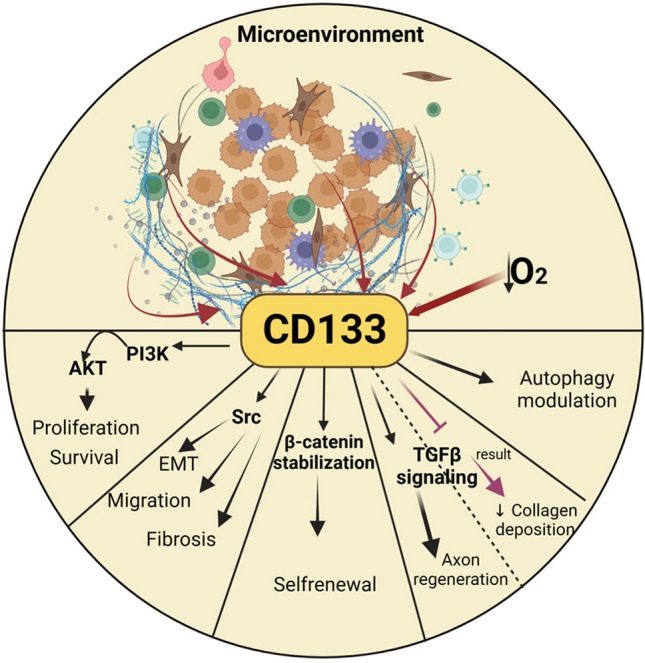

## Introduction

One of the primary methodologies employed to identify and select cancer stem-like cells is the utilization of cell surface markers, with CD133 emerging as one of the most robust markers across different cancer types. CD133 + cells demonstrate enhanced clonogenic capacity, particularly under anchorage-independent conditions, as well as tumor-initiating potential and resistance to chemotherapy compared to their CD133-negative counterparts within tumor. Moreover, CD133 expression has been linked to the co-expression of pluripotent genes, including Oct4, Sox2, Nanog, and c-Myc, which govern differentiation and self-renewal in pluripotent stem cells. This association has been correlated with long-term self-renewal and the ability of tumors to repopulate in a fraction of tumor samples [1]. Interestingly, the loss of CD133 in embryonic cells does not affect the expression of pluripotency genes or their capacity to differentiate into the three germ layers. Instead, it adversely impacts proliferation rates and the ability to evade apoptosis, according to findings from various studies on tumor cells. CD133 deficiency disrupts the coordinated regulation of key signaling pathways involved in tumorigenesis and apoptotic resistance, such as PI3K/Akt, WNT, AMPK, and P53 [2]. Similar outcomes have been observed in some cancer types [3, 4]

CD133 facilitates the establishment of signaling platforms at the plasma membrane by recruiting proteins involved in cytoskeleton rearrangement, protein trafficking, and signal transduction. These platforms are essential for regulating protrusion architecture, ciliary dynamics and activating signaling pathways that control cell proliferation, differentiation, migration, and cell survival. It is important to note that CD133 expression is not limited to the cell surface of cancer stem-like cells, suggesting a broader biological function in various cellular contexts. Moreover, the levels of CD133 are dynamic and can be enhanced or restricted depending on the cellular context, intrinsic state, and external stimuli from the surrounding niche. Its expression and processing can fluctuate throughout the cell cycle in different cell types [5, 6], increase under hypoxic conditions, or be repressed or enhanced in response to nutritional and cytotoxic stress.

In this review, we provide a comprehensive summary of our current knowledge regarding CD133's structure, post-translational modifications, and their impact on its function, subcellular localization, and protein stability. Additionally, we focus on how CD133 is regulated by changes in the tumor microenvironment, including oxygen levels, the extracellular matrix, tumor-associated cells, and chemotherapy, as drugs can impact the entire tumor microenvironment.

## CD133: Structure, Localization, and Posttranslational Modifications

CD133 (also called Prominin-1) is a pentaspan transmembrane glycoprotein widely used for stem and progenitor cell identification in several normal tissues and cancer stem-like cells of different types of cancer. CD133 is primarily associated with lipid rafts in the plasma membrane, forming part of a protein complex that regulates the structure and dynamics of protrusions (microvilli and primary cilia) [7–9]. Besides its cell surface localization, CD133 has also been found in the cytoplasm associated with endosomes as part of a cellular mechanism for recycling surface proteins and transporting them to other subcellular compartments. For example, in neuroblastoma, CD133 has been observed at the peri-centrosome region [10] and the nuclei in other cancers, such as rhabdomyosarcoma, hepatocarcinoma, breast cancer, non-small lung cancer, and colon-rectal cancer [11–15]. In addition, Rossi et al. (2019) reported that non-tumoral cells, such as endothelial colony-forming cells (ECFCs), characterized by lacking CD133 in their cell surface, exhibit CD133 intracellular expression, which was associated with revascularization potential after hind limb ischemia in contrast to mature endothelial cells [16]. The cytoplasmic and nuclear levels of CD133 have been associated with poor prognosis in non-small cell lung cancer and hepatocellular carcinoma [13, 14, 17], but further investigation is needed to understand its role in these subcellular compartments. Additionally, CD133 is secreted by cells in extracellular microvesicles and could potentially influence acceptor cells' phenotype and cellular state [18]. The different subcellular pools of CD133 could participate in specific biological processes such as proliferation, migration, autophagy, and chemoresistance in a cell context-dependent manner.

CD133 consist of five transmembrane domains, two extracellular loops susceptible to glycosylation, two smaller intracellular loops rich in cysteine, and a C-terminal domain. Mammals have at least 12 identified transcript variants of CD133, six of which differ in their C-terminal domain, potentially leading to interactions with different proteins and downstream signaling cascades [19]. While the C-terminal domain and its posttranslational modifications are crucial for the biological function and specificity of CD133 as a scaffold protein, posttranslational modifications in the extracellular domain are involved in CD133 cellular localization and stability.

Among the possible posttranslation modifications of CD133, N-glycosylation plays a critical role in protein folding, molecular trafficking, protein function, and protein stability. CD133 has nine predicted N-glycosylation sites [20], and although the loss of single-N-glycosylation site does not affect CD133 levels or delivery to the plasma membrane, the deficiency of all glycosylation sites impairs cell surface localization and results in endoplasmic reticulum (ER) retention, likely due to improper protein folding [20, 21] Furthermore, differential glycosylation of CD133 has been associated with cell differentiation rather than decreasing total protein, mRNA levels, or intracellular retention [22]. Different classes of N-glycans attached to the protein can modify its conformation, solubility, antigenicity, activity, recognition by glycan-binding proteins, and susceptibility to proteases [23]. This suggests that differential N-glycosylation of CD133 extracellular domain among tumor sub-populations may be related to specific functions, subcellular localization, and turnover. In line with this, Wei et al. (2022) reported that CD133 C-terminal domain interaction with DNMT1 (DNA methyltransferase 1) depends on CD133 glycosylation status in glioma stem-like cells (GSCs) [24]. High-mannose CD133 maintains GSCs in a slow-cycling state (quiescence) by blocking DNMT1 nuclear translocation. In contrast, induction of complex CD133 N-glycans through MAN1A (Mannosyl-oligosaccharide 1,2-α-mannosidase IA) ectopic expression promotes DNMT1 nuclear translocation and impairs long-term self-renewal and tumorigenesis [24]. The mechanism by which high-mannose N-glycan of CD133 regulates its interaction with DNMT1 remains unclear.

In addition, Liu et al. (2015) found that N-glycosylation at Asn548 of CD133 appears to mediate its interaction with β-catenin and downstream signaling. Mutation of the N-glycosylation at Asn548 reduced CD133-β-catenin interaction, β-catenin levels, and β-catenin signaling in hepatocellular cancer cell lines [20]. However, it is not specified whether the interaction is direct or mediated by other proteins such us HDAC6 (histone deacetylase 6). CD133 can associate with HDAC6 and β-catenin in a ternary complex that regulates the β-catenin stability, resulting in increased β-catenin co-transcriptional activity in ovarian and CRC cancer cell lines [25]. Interestingly Mak et al. (2012) showed that β-catenin interacts exclusively with a lower-molecular-weight form of CD133 and not with CD133 complex-glycans [25]. These findings suggest that N-glycosylation of one or more Asp residues and the type of glycans attached to the CD133 extracellular domain could alter the protein conformation and confers specificity to its intracellular domain, but further investigation is required.

### Regulation of CD133 Stability

In addition to glycosylation, there is evidence that CD133 can suffer other post-translational modifications, such as acetylation and sialylation on its extracellular domain. Acetylation of lysine residues (K216, K248, and K255) in the first extracellular loop of CD133 is involved in trafficking from the ER/Golgi to the plasma membrane but also ensures proper protein folding and maturation of the nascent protein [26]. Mak et al. (2014) reported that acetylation disruption in the ER lumen with CD133-K-to-R mutants and ATase1/2 (ER-based acetyltransferases) knockdown severely reduced CD133 surface localization and protein levels [27]. However, it is unclear whether CD133 lysine acetylation in the ER lumen is only a transient event to mark correct protein folding allowing the protein to move along the Golgi apparatus, where it could be deacetylated and complete its maturation. On the other hand, sialylation terminal modification of CD133 N-glycans contributes to protein stability, allowing CD133 to avoid lysosomal degradation [28]. However, it is also unclear how sialylation protects CD133 from degradation and how it could support CD133 function, considering that sialylation of glycoproteins could mediate cell–cell interaction, ECM-interaction, ligand-receptor interaction, and intracellular downstream signaling in several biological processes [29, 30].

Mak et al. (2012) reported that the CD133 protein is endocytosed and degraded via the lysosomal pathway when CD133-HDAC6 interaction is inhibited [25]. But, given that the CD133 intracellular domain does not seem to be an HDAC6 target, and even so, its deacetylase activity is required to maintain CD133 stability, these data suggest the existence of an intermediary protein, an HDAC6 target involved in CD133 regulation on the cell surface in CRC. Gao et al. (2010) found that HDAC6 regulates microtubule-dependent EGFR-endocytic trafficking and degradation through tubulin deacetylation. HDAC6 deficiency induces microtubule hyperacetylation, and consequently, the delivery of EGFR-containing endosomes to the lysosomal compartment is accelerated [31]. Also, HDAC6 could mediate pERK-microtubules association essential to maintain ERK activity and lung cancer cell proliferation via α-tubulin deacetylation [32]. These data suggest that HDAC6 might modulate CD133 association with microtubules close to the cell surface, CD133 endocytic traffic, and subsequent degradation, and even the duration of endosomal CD133-mediated signaling.

Aside from the cell's intrinsic state and cell-autonomous features that can regulate CD133 expression, changes in the niche, such as oxygen concentration, glucose, and lactate levels, can modulate CD133 levels. For instance, CD133 stability is regulated in hypoxia by GLT8D1 (Glycosyltransferase 8 Domain Containing 1), a transmembranal glycosyltransferase highly expressed under hypoxia by the HIF1α transcription factor [33] GLT8D1 co-localizes with CD133 in microvilli of glioma stem-like cells, where it glycosylated CD133, hindering its degradation via the endosomal-lysosomal pathway. Also, Liu K et al. (2022) reported that GLT8D1 mutant with glycosyl transferase activity deficiency can partially rescue GLT8D1 depletion-induced CD133 degradation, suggesting that even GLT8D1 physical association with CD133 irrespectively of its activity, decreases CD133 degradation.

Regardless of oxygen levels, cancer cells have an increased glycolytic metabolism compared to normal cells, which favors proliferation and cell survival. Indeed, cancer-stem-like cells (CSCs) of different types of tumors exhibit a higher expression of glycolytic enzymes resulting in enhanced glycolysis compared to more differentiated cancer cells. Some of these enzymes even can participate in other tumoral functions, including cell cycle progression, tumor immune evasion, stemness, and epigenetic regulation [34, 35]. Recently Wang et al. (2022) demonstrated that HK2 (hexokinase 2) enzyme modulates the CD133 stability through direct binding without subverting CD133 mRNA [36]. Its interaction promotes the recruitment of deubiquitinase ubiquitin-specific protease 11 (USP11), which inhibits CD133 polyubiquitination and its proteasomal degradation in small cell lung cancer (SCLC). By contrast, differentiated SCLC cells showed a lower CD133-USP11 interaction than CSCs, which indicates another CD133 differential regulation between intratumoral subpopulations apart from gene and epigenetic regulation. As well, PFKFB (6-phosphofructo-2-kinase/fructose-2,6-bisphosphatase-3), a key glycolytic enzyme, has been reported to maintain CD133 expression, promote cell cycle progression, cancer stemness, and apoptosis evasion in some cancers via dependent and independent way of its glycolytic activity [37–39]. Lactate, as a subproduct of glycolysis, contributes to enhancing CD133 levels in hepatocellular cancer [40], oral squamous cell carcinoma [41] and colorectal carcinoma cells [42]. High glycolytic cells in the tumor release lactate to the extracellular space, which can be taken by neighbored tumoral or stromal cells through mono-carboxylic acid transporters (MCTs). Lactate conversion into pyruvate in tumoral cells supplies mitochondrial oxidative phosphorylation, serving as an energy source but also as a metabolite that can influence upstream signal activation directly or indirectly through ROS generation, histone lactylation and histone acetylation that could regulate expression of pluripotent genes such as Oct4, Sox2, c-Myc, and also could improve CD133 transcription and acquisition of cancer-stem like features [43].

### CD133 Epigenetic Regulation

CD133 protein is coding by the PROM1 gene, whose expression is controlled by at least six alternative promoters in a tissue-dependent manner, three of which include several CpG sites highly susceptible to methylation. In vitro methylation of P1, P2, and P3 promoters suppresses their activity [44–46], indicating that promoter methylation status is associated with the regulation of CD133 expression. The hypomethylation state of these promoters (P1, P2, and P3) has been positively correlated with increased expression of CD133 in several cancers [47–50]. The DNA methylation status can change in dependence on the surrounding niche. For instance, TGFβ and BMP proteins, members of the TGFβ family secreted by tumoral cells or/ and other cells present in the tumor, have bivalent functions depending on the type of cancer and progression stage, displaying tumor suppressor or oncogenic role. In glioblastoma, whereas TGFβ promotes the growth and maintenance of cancer-like stem phenotype, BMP proteins induce differentiation and apoptosis [51]. Both types of proteins regulate CD133 expression, a robust marker of glioma-initiating cells. TGF-β stimulation induces CD133 expression through the downregulation of DNA methyltransferase 1 (DNMT1) and DNMT3β expression, leading to significant demethylation of the CD133-promoter 1(P1) [52]. SB431542, a TGFβ receptor I inhibitor (ALK5/ALK4 inhibitor), decreased the proportion of CD133 + subpopulation in glioma spheres and the sphere-forming ability [53]. By contrast, BMP4 shows the opposite effect by inducing PROM1 P1 promoter methylation, leading to its reduced activity and low CD133 expression. BMP4 upregulates the paired related homeobox 1 (PRRX1), a transcription factor involved in the development of the nervous system. Whereas both isoforms of this protein can bind to the PROM1 promoter, only the long isoform (PRRX1A) with OAR (otp, aristaless, and rax) domain in its C-terminal region (PRRX1A) interacts with DNMT3A and induces PROM1 promoter P1 methylation. Both PRRX1A and DNMT3A silencing increase CD133 + positive subpopulation even in the presence of BMP-4 and enhance their tumorigenic capacity [54].

Moreover, the poor association between methylation status and CD133 mRNA levels found by some authors suggests the existence of other mechanism that cooperates with DNA methylation on CD133 regulation, such as control of chromatin condensation by histone modifications even when DNA methylation was absent [55]. The balance between active histone modifications (H3K27ac and H3K4me3) and inhibitory histone marks (H3K27me3 and H3K9me3) can be crucial in PROM1 transcription [47, 55, 56]. High levels of H3K9me2 repressive mark in PROM1 P1 promoter inhibit its activity and CD133 expression in glioma cells, whereas G9a (histone-lysine *N*-methyltransferase, H3 lysine-9 specific 3) inhibition by bix01294 increases CD133 and Sox2 expression resulting in improved sphere-forming efficiency [57]. Also, CD133 mRNA overexpression was observed after treatment with trichostatin A, a histone deacetylase (HDAC) inhibitor in primary prostate epithelial cultures [53, 55]. CD133 upregulation correlated with increased levels of H3K27ac and H3K4me3 on P1, P2, and P3 promoters as well as high levels of histone acetyltransferases P300/CBP-associated factor (PACF) and H3K27me3 demethylase (HDM6B) along with reduced levels of H3K4me3 demethylase (LSD1) in LoVo colon-rectal cancer cell line that overexpressed Aldehyde oxidase 1 (AOX1) [56].

Furthermore, PROM1 promoter activity is controlled by cis-regulatory elements such as enhancers in acute lymphoblastic leukemia (ALL). The MLL-AF4 fusion protein, one of the most common rearrangements of the MLL (Mixed Lineage Leukemia) gene that promotes leukemogenesis, regulates promoter-enhancer interaction between PROM1 and its nearby gene TAPT1 by recruitment of DOT1L (Dot1-like, histone H3K79 methyltransferase) and subsequent increase of histone H3K79me2/3 on intragenic enhancer elements present in both genes, which in turn potentiate transcription of PROM1 and TAPT1 genes in some MLLr leukemias [58]. PROM1 promoter repression by polycomb repressive complex 2 (PCR2) in CD133-negative leukemia cells impair the interaction between the PROM1 promoter and the intragenic PROM1 and TAPT1 enhancers, explaining the absence of CD133 expression [58]. In addition, restriction of CD133 expression to immature CD34 + stem/progenitor cells in normal fetal and adult bone marrow cells correlates with relaxing chromatin in the PROM1 locus and MLL binding to the PROM1 promoter. In contrast, mature cells exhibit high levels of H3K27me3 mark on the PROM1 promoter, consistent with its repression by PCR2.

## CD133 Biological Function (Signaling Pathways)

### CD133-PI3K/Akt Signaling

PI3K/Akt signaling is one of the main pathways that mediate the phenotypic effects correlated with CD133 expression in the cell surface of tumoral cells. Akt signaling promotes proliferation, survival, migration, invasion, and chemo-resistance by activating its downstream effectors in different cell types [59, 60]. CD133 mediates Akt activation by recruitment of the p85 regulatory subunit of PI3K to its C-terminal domain previously phosphorylated by Src kinase at Y828 residue, a critical event to allow its interaction with p85 [61]. Once active, PI3K converts PIP2 (phosphatidylinositol 4,5-biphosphate) into PIP3 (phosphatidylinositol 3,4,5-triphosphate), leading to Akt activation via PDK1 (3-phosphoinositide-dependent protein kinase 1). Y828F/Y858F mutants and pharmacological inhibition of Src (using PP2) significantly inhibit the CD133-p85 interaction and Akt activation in glioma cells and then its self-renewal and tumor-initiating capacity [61].

Given that CD133 phosphorylation is crucial to mediate PI3K/Akt signaling, its dephosphorylation is an event that could restrict its function; Shimozato et al. (2015) identified that PTPRK (receptor-type protein tyrosine phosphatase K) interacts with CD133 and negatively regulate Akt activation [62], whereas its knockdown enhances tumor growth and survival due to CD133 ectopic expression in CRC under nutritional and cytotoxic stress [63, 64]. Low expression of PTPRK correlates with poor prognosis in patients with CD133 expression, suggesting that PTPRK might abrogate CD133 pro-oncogenic function in CRC. Also, Kim et al. (2018) showed that PTPRF (receptor-type protein tyrosine phosphatase F) negatively modulates cell adhesion, migration, anchorage-independent growth, and cell survival, and its silencing recovered partly the reduced sphere growth induced by CD133 knockdown [65]. Although PTPRF might reduce CD133 function by direct interaction and subsequent tyrosine dephosphorylation, also it can dephosphorylate Src and decrease its activity leading to CD133-dependent and independent downstream signaling inhibition [65, 66]. CD133 promotes TM4SF5 (transmembrane 4L six family member 5) expression through the Akt/β-catenin axis, and TM4SF5 enhances CD133 function by facilitating Src activation [67] and altering PTPRF stability or even its activity [65]. CD133-TM4SF5 in hepatocellular cancer context establishes a positive feedback loop to maintain proliferation and survival.

Regardless of the ability of CD133 to recruit p85 and facilitate downstream Akt activation, CD133 can also modulate Akt activation through receptor tyrosine kinases stabilization. For instance, CD133 stabilizes EGFR (epidermal growth factor receptor) in the plasma membrane by direct interaction. CD133 avoids EGFR endocytosis and sustains EGFR-mediated Akt activation [68, 69]. CD133 silencing decreases HER3 levels and suppresses EGFR and HER2 activation without disturbing their gene transcription in CRC cell lines [70]. CD133 silencing reduces proliferation, clonogenic capacity, migration, cell invasion, and resistance in CRC in part through decreasing glucose uptake as a result of the low GLUT1 expression because of impaired HER3/Akt pathway involved in protein synthesis via mTOR and mRNA stability, probably through the regulation of RNA-binding proteins [70, 71]. Enrichment RNA analysis showed that CD133 + cells in gastric cancer have high expression of RNA-modifying enzymes that modulate RNA decay, mRNA translation, pre-mRNA processing, and RNA export compared to CD133-negative cells [72]; however, the precise role of CD133 in mRNA processing is unclear.

### CD133- Src Signaling

Aside from Src playing an indispensable role in the CD133 function on PI3K/Akt signaling, CD133-Src interaction favors Src activity. Whereas Y828 in the C-terminal domain of CD133 is the target of Src, the region between 845 and 857 amino acids seems essential for its interaction with Src and Src subsequent activation [73]. Although it is unclear how CD133 induces or amplifies Src activity, the binding of CD133 p-Y852 with the Src SH2 domain could trigger Src conformational change that promotes its activation (by autophosphorylation) [74]

CD133-Src signaling may mediate migration, invasion, and metastasis in cancer. CD133-Src-FAK axis promotes cell migration in the SW620 CRC cell line only under serum starvation, whereas CD133 and Src do not seem to interact under optimal culture conditions. CD133 deficiency decreased Src and FAK activation, causing reduced migration ability under serum starvation [73]. These data suggest that the articulation of signaling pathways by CD133 could be context-dependent. Furthermore, CD133 knockdown or Src inhibition abrogates the increasing levels of N-cadherin and vimentin induced by CD133 overexpression in head and neck cancer cell lines, whereas promoting epithelial morphological acquisition characterized by E-cadherin and CK18 epithelial marker upregulation [75]. Also, CD133 silencing has been directly correlated with epithelial-mesenchymal transition (EMT) inhibition in gastric cancer (downregulation of Snail, Slug, N-cadherin proteins) [76] and with suppressed invasion and metastasis in pancreatic cancer [77].

Src upregulation can promote tumor growth and drive metastasis by linking to several pathways [74, 78]. Src-mediated YAP/TAZ activity and its target genes in melanoma and breast cancer drives tumor growth and metastasis [79]. Oh HT et al. (2022) demonstrated that CD133 regulates TAZ levels and nuclear localization via Src activation without altering YAP levels in cholangiocyte cells after ductal injury. CD133 knockdown and Src inhibitor reduced TAZ levels and fibrosis inducers such as CTGF (connective tissue growth factor), CYR61 (Cysteine Rich Angiogenic Inducer 61) and TGFb1 (Transforming growth factor beta-1) in cholangiocyte organoids and the HCT116 CRC cell line [80]. Also, YAP/TAZ activity promotes EMT markers expression [38] and TAZ is required to sustain self-renewal in breast cancer stem-like cells [81] and intestinal tumor initiation [82]. This evidence suggests that CD133-src signaling via TAZ stabilization could participate during cancer initiation and aggressive phenotypes in different cancer types by promoting stiffing stroma, EMT acquisition, and maintaining cancer stem-related traits.

On the other hand, CD133-Src could mediate cell proliferation through cdc42 inhibition via phospho-caveolin-1 binding. Aside from participating in actin polymerization during the formation and maintenance of filopodia, Cdc42 promotes the destabilization of a group of subunits of the BAF complex, leading to BAF complex assembly inhibition [83]. BAF complex is an ATP-dependent chromatin remodeling complex that controls gene expression in cancer [84] and during mammalian development (neural, heart, muscle, and even stem pluripotency). In Ewing sarcoma, EWS-FL1 recruits BAF complex to its target genes to induce their transcription, thereby BAF complex disassembly by Cdc42 activation reduces EWS-FL1 target gene expression and its participation in proliferation and tumor progression [83].

### CD133- β-catenin Signaling

Previously, we mentioned that CD133 enhances Wnt signaling by increasing β-catenin levels through GSK3 inhibition mediated by Akt, which allows to β-catenin avoid β-TrCP polyubiquitination and subsequent degradation. Apart from phosphorylation, other posttranslational modifications regulate the Wnt/β-catenin pathway, such as acetylation, sumoylation, and ubiquitination affecting activity, protein–protein interaction, protein stability, and subcellular localization [85, 86]. Mak et al. (2012) showed that CD133 interaction with HDAC6 promotes β-catenin lysine49 (K49) deacetylation, blocking phosphorylation-dependent degradation. CD133 or HDAC6 downregulation results in β-catenin destabilization and reduced β-catenin/TCF transcriptional activity, which correlates with decreased proliferation and cell differentiation in Caco2 and OVCAR-8 cell lines [25]. Also, TCF-LEF-binding sites are present in the PROM1 gene promoter [87] and inhibition of CBP-β-catenin interaction downregulated CD133 expression in hepatocellular cancer [88], which suggest a feedback loop between CD133 and β-catenin signaling.

In adult tissues, CD133 promotes the long-term self-renewal of pancreatic progenitors in part by increasing the β-catenin/TCF signaling in response to RSPO1 stimulus, a Wnt agonist, by constraining β-catenin phosphorylation mediated by GSK3β. Curiously, Tremblay et al. (2019) and Brossa et al. (2018) found that CD133 can be physically associated with E-cadherin and β-catenin in pancreatic and renal progenitors. Although this complex might limit β-catenin cytoplasmic degradation, the precise CD133 role is undetermined [89, 90]. Frequently, the β-catenin bound to E-cadherin in adherent junctions has been considered sequestered and unavailable for signaling. Nevertheless, the E-cadherin-β-catenin complex stability is regulated by phosphorylation in response to external stimulus [91]. For instance, growth factor receptors and Src phosphorylate β-catenin, causing its release from the plasma membrane and subsequent nuclear translocation, wherein β-catenin acts as a co-transcriptional activator [92]. Considering that CD133 also can interact with Src and EGFR, CD133 could coordinate the phosphorylation of β-catenin in the membrane upon specific stimuli and promote its cytoplasm accumulation. In addition, E-cadherin loss in mouse spermatogonial progenitor cells reduces β-catenin levels, resulting in less β-catenin to signaling and increased expression of differentiation markers [93]. In cancer, although E-cadherin downregulation is associated with beta-catenin nuclear localization and increasing β-catenin/TCF transcriptional activity, several reports do not support such correlation and β-catenin signaling requires additional events to assure its activation [94]. CD133 with E-cadherin might be hubs that coordinate the intracellular response to changes in the microenvironment and fine-tune the activation of the pathway in dependence on the cellular state.

### CD133- Smad Signaling

TGFβ signaling controls various cellular processes during embryonic development and tissue homeostasis in adult tissues. In cancer, TGFβ signaling frequently exerts a tumor suppressor function during the early stages of tumorigenesis, but its activation favors cell invasion and metastasis in advanced tumors. Recent studies have found that CD133 regulates TGFβ signaling during cell repair processes in neurons and liver cells. CD133 can interact with members of the signaling cascade and activate or contribute to TGFβ signaling inhibition in dependence on the cell type.

In mature neurons, CD133 low expression correlates with a less axon regeneration capacity than neurons in the early stages of development. Despite of CD133 downregulation in DRG (dorsal root ganglion) mature neurons, CD133 is required for optimal axon regeneration in mice after sciatic nerve injury, and its overexpression improved axon growth even in non-permissive microenvironments [95]. CD133 controls cholesterol metabolism in injured neurons through R-Smads transcription activity, and cholesterol depletion favors axon regeneration [95, 96]. Mechanistically, CD133 interaction with the ALK4 receptor enhances smad2/3 downstream signaling, leading to the downregulation of gene-associated cholesterol biosynthesis. High cholesterol levels due to pharmacological CD133 and ALK4 inhibition overturned axon regeneration enhancement induced by CD133 upregulation in both embryonic DRG neurons and adult nerves of mice treated with adeno-associated virus-mediated PROM1 gene delivery in a model of sciatic injury [95]. How CD133 interacts with ALK4 and increase the TGFβ ligands signal requires further investigation.

By contrast, CD133 negatively regulates TGFβ signaling by promoting Smad7 stabilization in hepatocytes after bile duct ligation in mice as a model of fibrosis induction. Smad7 is an inhibitory Smad that induces TGFβ receptor I inactivation and degradation through different protein complexes recruitment, hindering Smad2/3 phosphorylation and downstream signaling [97]. CD133 bound to Smad7 avoids Smad7-SMURF interaction, thereby abolishing Smad7 ubiquitination and subsequent degradation [98]. Whereas CD133 in hepatocytes mitigates collagen deposition and apoptosis during liver fibrosis, other authors have reported that the CD133-Src-TAZ axis has an opposite effect in bile ductal cells, promoting fibrosis inducers secretion, such as TGFβ1 and CTGF that activate hepatic stellate cells to produce extracellular matrix in a model of diet-induced fibrosis [80]. Together, this evidence reinforces the idea that CD133 modulates different responses by recruiting a wide range of proteins in a cell-context manner. It will be interesting to know if CD133 might regulate canonical and non-canonical Smad signaling during cancer progression.

### CD133- Hedgehog Signaling

Hedgehog signaling is a cilium-dependent pathway in non-tumoral cells [99] involved in embryonic development, tissue homeostasis, and regeneration, and its deregulation contributes to cancer development [100]. In normal cells, primary cilium allows the spatial regulation of canonical Hedgehog pathway in response to stimulation with Hh ligands, which is necessary for stem cell activation in various tissues [101–103]. In the absence of Hh ligands, the PTCH1 (Patched 1) receptor localizes at the base of the primary cilium, wherein inhibits and excludes Smoothened (Smo) from cilium through an unclear mechanism. PKA (protein kinase A) and SuFu (suppressor of fused) repress Gli transcription factors at the tip of the cilium and promote their cleaved into Gli-R, which acts as a transcriptional repressor. Hh ligands inhibit PTCH1 and, in turn, promote SMO accumulation in the cilium, wherein activated SMO releases Gli proteins from SuFu repression and avoids Gli proteolysis; then, Gli can enter the nucleus and activate gene target expression [104].

Associated CD133 to membrane microdomains can orchestrate signaling in the cilium of the different types of cells. Singer et al. (2019) demonstrated that CD133 is essential to coordinate ciliary dynamics with stem cell activation in cervical incisor loop epithelium (CLE) as a model of stem cell activation. CD133 deficiency impairs self-renewal, transient-amplification (AT) cells proliferation and differentiation, which correlated with a decreasing expression of Gli1-3 in AT cells and loss of Glis2 (Gli-similar 2) localization in the primary cilium of stem population of CLE [9]. Whereas Gli1-3 proteins are the most common terminal downstream effectors of Hedgehog signaling, how Hh ligands could regulate Glis proteins is unknown. Nevertheless, the high homology between their DNA binding domains suggests that they can compete for the same DNA binding site and likely mediate opposite transcriptional activity; indeed, Glis2 appears to play mainly as a transcriptional repressor [105]. Singer and colleagues reported elevated Glis2 levels in AT cells and a minor extent in stem cells in CLE, which correlate with CD133 expression. CD133 seems to control Glis2 localization in response to the Hh ligands; in its absence, CD133 restricts Glis2 to the primary cilium in stem cells to maintain stem cell quiescence; in response to Hh stimulation, CD133-Glis2 complex translocates to the nucleus in a time-dependent manner, and acts as transcriptional repressor of genes involved in stem cell maintenance and self-renewal such as STAT3 during commitment and cell differentiation [9]. Although Glis2 represses STAT3, especially in TA cells, the CD133 function in the nucleus as a partner of Glis2 is unclear, and further investigation is also required to clarify the CD133 function in stem cells and during the transition to TA cells. In addition, the question arise whether CD133 could regulate Glis activity in tumor cells given Glis link with some cancers [106], and how Hh ligands could mediate its function.

### CD133 and Autophagy

Autophagy is a degradation/recycling mechanism designed to control protein expression levels, the turnover of damaged organelles, and long-lived proteins to maintain cellular homeostasis [107, 108]. The targeted proteins or components are encapsulated in vesicles and degraded by lysosomal enzymes. The sub-products of degradation are utilized for the biosynthesis of new cellular components or to obtain energy [109]. Autophagy may increase as an adaptive response to stress conditions to enhance survival and re-establish cell homeostasis.

CD133 has become associated with autophagy upregulation in some cancers, such as hepatocarcinoma and glioma cells. CD133 enhanced tolerance to nutritional stress through increased glucose uptake and autophagy. CD133 translocates to cytoplasm and partially colocalizes with LC3-II foci under glucose starvation, whereas CD133 loss attenuates autophagosome formation [110, 111], which suggests that CD133 could participate in autophagy induction and ultimately undergoes lysosomal degradation, but how specifically CD133 contributes to autophagosome assembly is undetermined. Izumi et al. (2022) found that CD133 is preferentially downregulated in the cytoplasm by p62-mediated selective autophagy/lysosomal degradation to control CD133 levels in neuroblastoma cells that exhibit high basal autophagy activity [112].

Recent studies indicate that CD133 could contribute to different processes and exert contrast functions in more primitive cells such as stem/progenitor cells and cancer stem-like cells, where autophagy increasing has been related to differentiation. Izumi et al. (2019) found that CD133 function and localization are regulated by its phosphorylation state. While Src phosphorylation appears to be necessary to maintain CD133 at plasma membrane localization and mediate CD133-mediated PI3K/Akt signaling under higher availability of mitogens and nutrients, non-phosphorylated CD133 is endocytosed and in dependence on the cellular state can be targeted to degradation or transported to the pericentrosomal region through HDAC6/dynein- based traffic system, in which could mediate other functions and subsequently be recycled. Indeed, CD133 traps GABARAP in the pericentrosomal zone and avoids GABARAP-mediated ULK1 activation (by binding), attenuating autophagy initiation [10]. In line with that, CD133 silencing in SK-N-DZ and Huh-7 cells increased ULK1 kinase activity and autophagosome formation. Surprisingly, CD133 knockdown in SK-N-DZ enhanced neurite outgrowth upon differentiation conditions related to high autophagy induction. These data suggest that CD133-pericentrosomal inhibits cell differentiation in medulloblastoma cancer cells through autophagy modulation under basal and nutritional stress conditions. Disturbed basal autophagy by increasing or depletion (serum starvation, Rapamycin) alters stemness and promotes differentiation and/or senescence [113]. Under this argument, CD133/GABARAP axis and even CD133/Akt/mTOR axis might maintain basal autophagy levels below a threshold that do not compromise cellular identity and functionality.

Furthermore, Izumi and colleagues (2022) demonstrated that unequal CD133-pericentrosomal distribution during asymmetric division contributes to heterogeneity autophagy activity; the daughter cell with CD133 high-pericentrosomal localization maintains low basal autophagy activity and stem-like treats, probably by reinforcing β-catenin nuclear localization and co-transcriptional activity. In contrast, the other daughter cell with less CD133 expression exhibits high basal autophagy activity, and β-catenin remains on the cell surface [112]. Tumoral cells subpopulations with a broad spectrum of autophagy activity and CD133 distribution might confer adaptive capacity upon stress-inducing conditions such as cytotoxic insult and starvation.

### Maintenance and Formation of Protrusions

Because of the predominant cellular localization of CD133 in microvilli and primary cilium, its loss or dysfunction by mutations cause degeneration of the outer segment of retinal photoreceptor cells in vertebrate and invertebrate animal models due to loss of ciliary structure in this cell type. Although other functional defects have not been associated with CD133 deficiency, likely because of compensation performed by co-expression of prominin-2 in other tissues, CD133 loss could be related with other ciliopathies beyond retinal degeneration [114–116].

At the molecular level, CD133 association with cholesterol in cytoplasmic membrane determines its retention in protrusions rich in cholesterol [117], while its interaction with gangliosides in the membrane can affect phospholipid composition driven by PI3K activation at the inner surface of the plasma membrane, and also recruitment of other intracellular proteins involved in the structural maintenance of microvilli and other membrane extensions [116, 118]. Point mutations in its ganglioside (GM1)-binding site in the N-terminal domain increases its interaction with the p85 regulatory subunit of PI3K and its downstream Arp1/2 complex activation that participate in actin-cytoskeleton rearrangement in polarized epithelial (canine kidney cells) and fibroblast-like cells, leading to the formation of branched microvilli or microvilli with knob-like morphology [115]. Y819F/Y828F mutants that impede carboxy-terminal phosphorylation impaired protrusions elongation (microvilli and primary cilia) and overturn the GM1-binding site mutants effect on microvilli architecture [116, 119].

In contrast, Hori et al. (2019) reported that PI3K activity and CD133 Y819/Y828 phosphorylation was not necessary to mediate CD133 function on fiber formation on the plasma membrane in cells of retinal pigmented epithelium (RPE-1 cells). Nevertheless, the KLAKY motif localized between 814 and 818 amino acids in the carboxyl-terminal region was essential and suggests that CD133 participates in maintaining structure and fiber biogenesis of membrane extensions, inducing different downstream signaling [120]. RhoA/ROCK was required to mediate CD133 formation fibers in CD133 overexpressing RPE-1 cells, but no physical interaction was detected. These data suggest a missing intermediary protein that probably triggers RhoA activation; in this case, CD133 might facilitate its recruitment at the membrane point.

In addition, CD133 regulates the assembly-disassembly dynamic of primary cilium through its interaction with Arl13b or HDAC6 in dental epithelial stem cells. Arlb13b is a member of the RAS small GTPase family involved in protein traffic in the cilium. Arlb13b loss along axoneme could impair protein traffic and disturb cilium elongation [9, 121] in stem cells, whereas HDAC6 induces disassemble of primary cilium through tubulin deacetylation when stem cells transition into transitory amplifying cells.

## CD133 Modulation by Microenvironment

### Regulation by Hypoxia

Several studies have reported that CD133 is upregulated under hypoxic conditions that emulate the low oxygen supply in the core of overgrown tumors and tissular regions with low microvessel density and leaky capillaries, which limit oxygen delivery. Cancer cells exhibited high tolerance to decreasing oxygen levels because they upregulate the expression of the Hypoxia-inducible Factors (HIFs) 1/2α that coordinate the adaptive response to intratumoral oxygen gradients. Loss-of-function mutations of tumor suppressor genes involved in the oxygen-sensing mechanism and oncogenic growth factors signaling contribute to increased gene transcription and translation of HIF1/2-α isoforms even under normoxic conditions [122].

Whereas HIF-1α and HIF-2α could activate PROM1 P5 promoter through association with the ETS transcription factors (ETS binding sites in promoter), individual HIF silencing decreases slightly P5 promoter activity in CRC under normoxia. Only double knockdown (of both HIFs) decrease significantly promoter activity resulting in reduced levels of CD133 protein in the WiDr colon cancer cell line [123]. CD133 upregulation in hypoxic conditions has been reported in glioma, pancreatic and renal cancer in part by HIF-1α-dependent manner [124–126]. In addition, hypoxia promotes CD133 expression through HIF1/2 α-induced Sox2, Oct4, and Klf4 upregulation in several cancers [127–129]. HIF1/2 α can regulate these proteins through direct and indirect mechanisms. For instance, Covello et al. (2006) demonstrated that HIF2α, but not HIF1α, activate OCT4 promoters that contain hypoxia response elements (HRE) [130]. Both HIF1α and HIF2α indirectly could upregulate Sox2 by promoting mRNA stability. HIF1α/HIF2α induce ALKBH5 (alkB homolog 5, RNA demethylase) expression during hypoxic conditions that demethylate Sox2 mRNA (m6A-modified mRNAs), leading to Sox2/CD133 upregulation and maintenance of cancer stem-like traits [131]. In addition, Both HIFsα subunits might promote chromatin accessibility through the upregulation of proteins involved in epigenetic regulation (epigenetic readers, chromatin structure regulators), leading to improved gene transcription [132]. Hao et al. (2022) found that increased ATAD2 (ATPase family AAA domain-containing protein 2) expression by chronic hypoxia-HIF1α induction enhances CD133 and CD44 levels in lung cancer [133]. ATAD2 plays an essential role in histone dynamics by recruitment of epigenetic modifiers [134] and mediating histone turnover [135].

CD133-negative glioma cells transplanted into brain mice expressed high levels of HIF1/2α and concomitant expression of CD133, which indicate the strong effect of the tumoral microenvironment on CD133 expression levels and cell phenotype [136]. Double knockout of HIF1α and HIF2α produced a superior negative impact on CD133 expression, tumor growth, and increased mice survival compared to the effect of individual knockout. In line with that evidence, Wang et al. (2017) found hyperoxia (95% O2) promotes cell differentiation of glioma stem-like cells through negative regulation of HIF1α, resulting in CD133, Nestin, and CD15 reduction, and high levels of GFAP, a marker of astrocytic differentiation [137]. Also, hyperoxia induces apoptosis, impaired clonogenicity growth, and temozolomide responsiveness, the opposite effect triggered by hypoxia.

Furthermore, HIF-1α could regulate CD133 expression by enhancing Notch, Hedgehog, and Wnt signaling. These pathways control the balance between the maintenance of stem cell subpopulation, its activation, and differentiation status in several adult tissues. Their disturbance is associated with perturbed tissue homeostasis during oncogenesis. HIF1α and HIF2α compete to interact with NICD (Notch intracellular domain) and regulate its co-transcriptional function in dependence on oxygen levels. HIF1α stabilizes NICD by binding and promoting its transactivation activity leading to CD133 increasing levels under hypoxia; Notch inhibitor (GSI) depletes CD133 mRNA levels and promotes neuronal and astrocytic differentiation [138]. Also, Jagged1 (Notch ligand) treatment blunted the HIF1α reduction effect on CD133, CD44, and ALDH1 levels, sphere forming efficiency, invasion capacity, and proliferation of prostate cancer cell lines exposed to hypoxia [139]. Although HIF2α represses NICD activity essentially upon normoxia and mild hypoxia, HIF-2α participates in CD133 regulation and seems independent of Notch signaling in glioma cells [138]. Also, Hedgehog signaling inhibition using cyclopamine (Smoothened SMO receptor inhibitor) and GANT61 (GLI inhibitor), which inhibit different steps along the cascade pathway, abolished hypoxia-enhanced CD133, Oct4, Sox2, Nanog expression, EMT marker expression and invasion capacity of cholangiocarcinoma cancer lines [140]. HIF1α silencing abrogated Shh ligand expression and downstream signaling during hypoxia. In addition, although PROM1 (CD133) is a Wnt-target gene [87] and HIFαs appear to display a counterbalancing function on β-catenin/TCF transcriptional activity in response to oxygen levels [141, 142], it is unclear how the balance between HIF1α and HIF2α during hypoxia contribute to Wnt pathway and CD133 regulation mostly in cancer cells where Wnt pathway plays a crucial role. Miao et al. (2023) reported that hypoxia-induced β-catenin stabilization upregulated CD133 and Nanog expression in CRC cell lines, and that β-catenin knockdown reversed this effect [143]. Also, Mazumdar et al. (2010) found that HIF1α transactivates LEF1 and TCF1, leading to Wnt/β-catenin target genes transcription only in ESC (embryonic stem cells), but not in embryonic cell-derived neurons during hypoxia [144]. By contrast Kaidi et al. (2007) reported that HIF1α has a negative role in β-catenin/TCF transcriptional activity in CRC cell lines by competitive binding with TCF4, in which HIF-1α/β-catenin transactivates hypoxia responsive target genes to promote an adaptative response to low oxygen levels [141]. These findings suggest that at least HIF1α exhibits differential function in a cell-context-dependent manner.

**Fig. 1:**
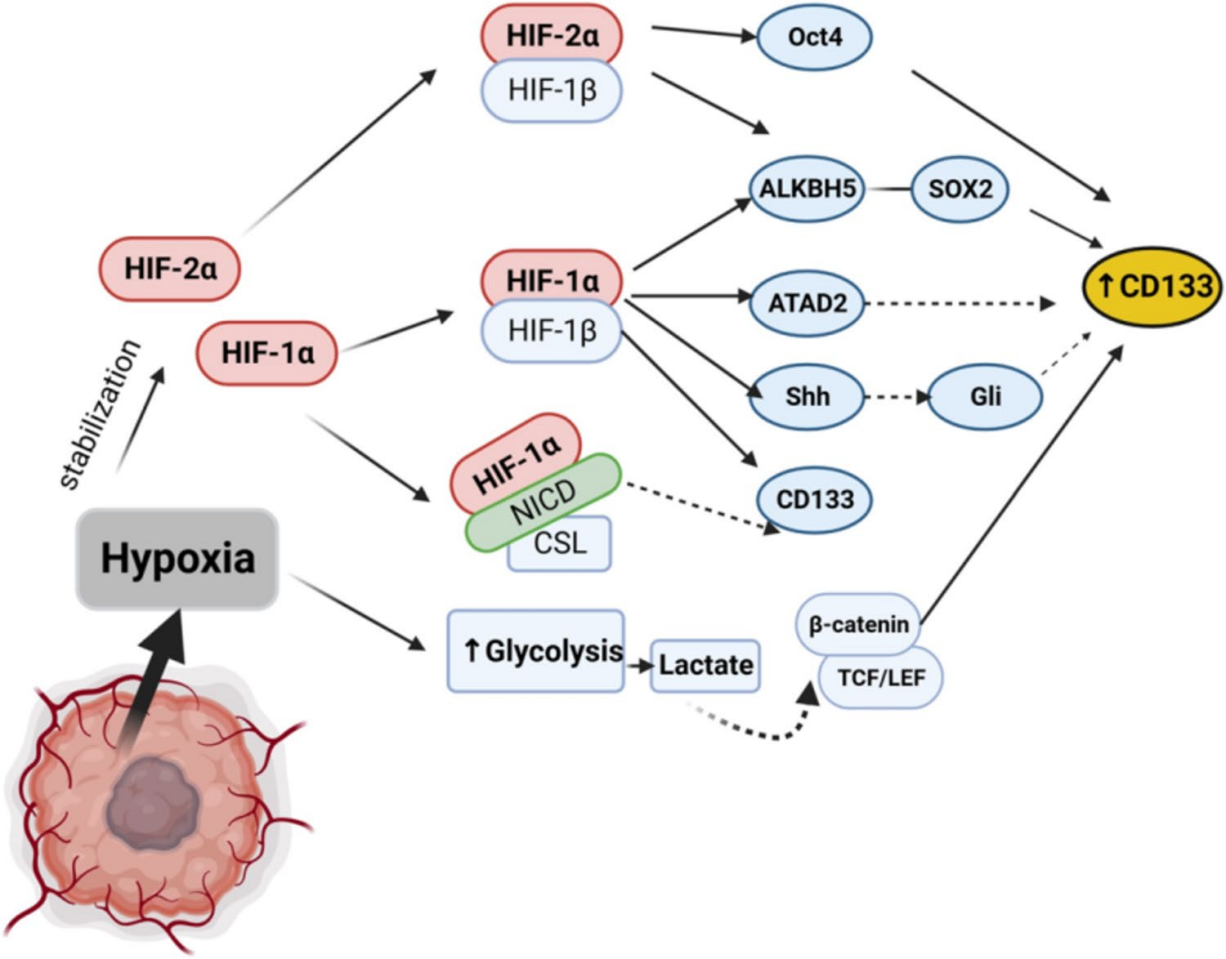
CD133 regulation in hypoxic conditions. Hypoxia-inducible Factors (HIFs) 1/2α upregulate the levels of Sox2 and Oct4, and also epigenetic regulators such as ATAD2 that favor gene transcription. HIF1α can stabilize NICD (Notch intracellular domain) in the nucleus and promote NICD/CSL transcriptional activity leading to improve CD133 expression as well as promoting Hedgehog signaling through improved Shh ligand. Also, hypoxia can promote β-catenin stabilization via increased glycolysis and lactate release leading to β-catenin nuclear translocation and CD133 expression. Created with BioRender.com

Together, this evidence suggests that CD133 expression is synergically upregulated by different transcription factors in response to hypoxia, in which case HIFαs balance and HIF1α/HIF2α switch during chronic hypoxia could be determinants in the maintenance of CD133 + population and expansion (Fig. 1).

### Reactive Oxygen Species (ROS)

One of the particular characteristics of cancer is the rise in the physiological threshold of reactive oxygen species (ROS) because of high cell proliferation, low oxygen availability, endogenous immune response (inflammation), and antineoplastic therapy that boosts ROS production. In addition, ROS as second messengers are essential for the local and transient regulation of signaling cascades that mediate several cellular functions. Both persistent overproduction and underproduction beyond the thresholds could compromise cell viability and other cellular processes [145].

Tumoral cells can display adaptive mechanisms that restrict ROS levels to tolerable limits, such as increased expression of antioxidant enzymes, restriction of intracellular ROS production (from mitochondria, from NADPH oxidases) by metabolic reprogramming, and the reduction of hydrogen peroxide transporter channels, among others [146]. For instance, CD133 expression correlated with superoxide dismutase (SOD) and cystine/glutamate transporter xCT, crucial to anion superoxide conversion to hydrogen peroxide (less reactive and diffusible) and glutathione levels maintenance, respectively [147, 148]. CD133 depletion negatively impacts xCT induction in response to H_2_0_2_ treatment and sensitizes tumoral cells to conventional antineoplastic drugs, but how CD133 promotes an xCT increase is undetermined [148]. Also, Zheng et al. (2020) found that Aquaporin 9, a water channel that facilitates H_2_0_2_ transport from the extracellular to intracellular space, is downregulated in HCC and that its overexpression disturbed cancer stem-like cell activation and CD133 expression as well as other cancers stem cell markers [149]. AQP9-mediated intracellular ROS disturbed β-catenin-TCF4 association and enhanced β-catenin-FOXO3a interaction, leading to Wnt-target genes downregulation. FOXO transcription factors are known to be regulated by ROS intracellular levels; its activation could determine the cell cycle exit under unfavorable conditions [150, 151]. Consistent with this, NAC treatment rescued β-catenin-TCF4 association and CD133 expression in HCC. In addition, IGF downregulates AQP9, whereby restrains intracellular H_2_0_2_ levels and promotes cancer stem-like properties.

**Fig. 2:**
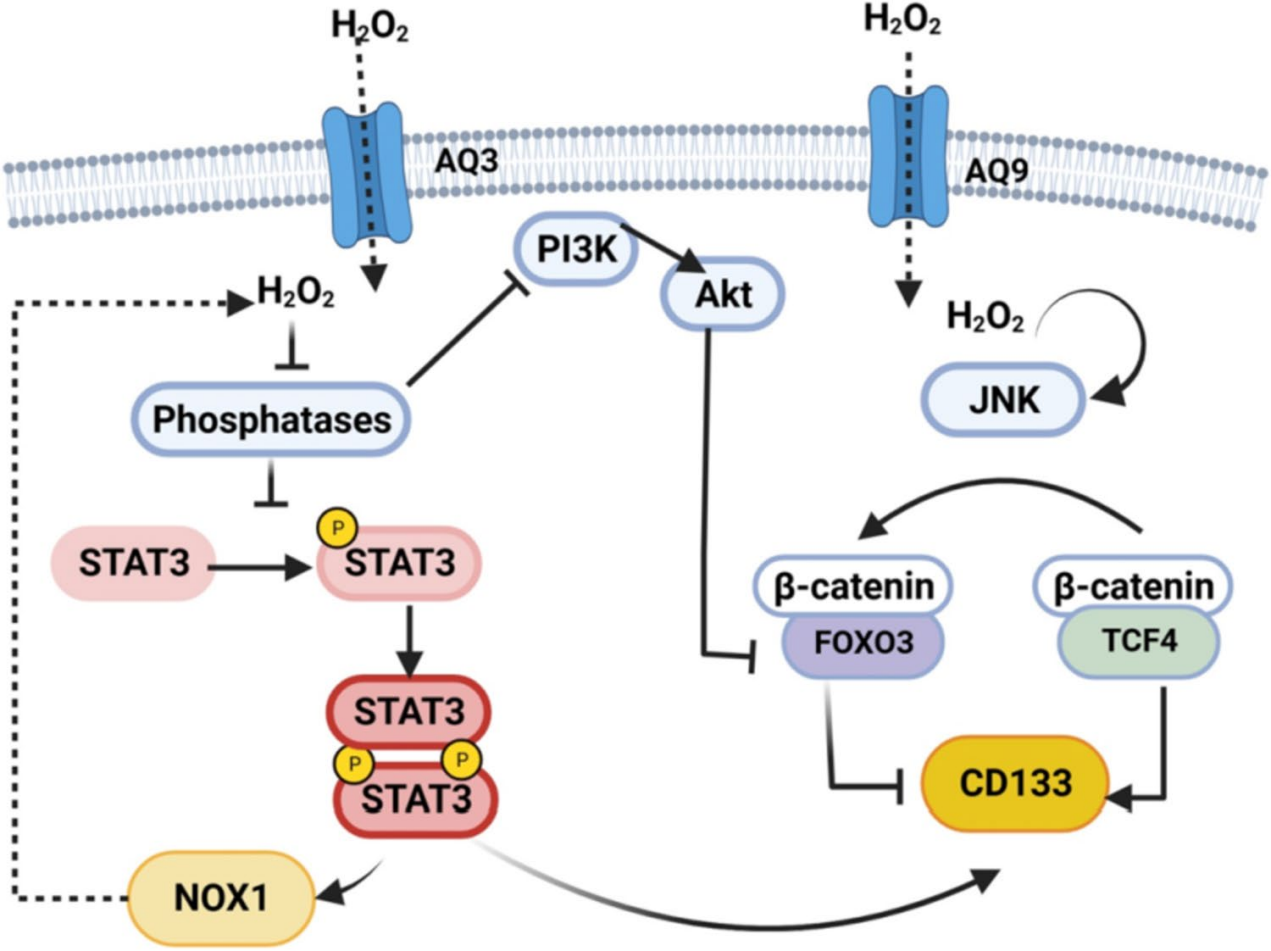
ROS modulates CD133 levels in a context-dependent manner. Increased ROS species by aquaporin (AQ 3 y AQ9) channels can positively or negatively regulate CD133 expression because of its role in signaling modulation. ROS could favor JNK activation and downstream signaling. JNK induces FOXO3a release from 14–3-3 protein and subsequently its entry to the nucleus, wherein it competes with TCF4 to bind to β-catenin, leading to CD133 downregulation. By contrast, ROS also can improve CD133 expression by enhancing STAT3 and PI3K signaling. Created with BioRender.com

By contrast, Wang et al. (2019) reported that Aquaporin 3 (AQP3) exhibited an opposite effect on CD133 regulation and stem-like properties in HCC [152]; nevertheless, in this study, Aquaporin-mediated H_2_0_2_ transporting was not evaluated, and AQP3 could have functions independent on mediated H_2_0_2_ intracellular levels, such as glycerol uptake and even its cytoplasmic localization could be associated with an intracellular role [153]. AQP3 promotes STAT3 activation, nuclear translocation, and PROM1 promoter activation in HCC [152]. In several models, ROS promotes STAT3 activation probably by thiol oxidation in the active site of tyrosine phosphatases, a negative regulator of STAT3 activity [154]. Activated STAT3 transactivates NOX1, producing a feedback loop that sustains REDOX signaling (STAT3 and PI3K/Akt activation) involved in migration, cell proliferation, and stem-like properties [155, 156] (Fig. 2).

### Extracellular Matrix

As part of the cellular microenvironment, the extracellular matrix (ECM) provides mechanical support and plays a critical role in cellular behavior and phenotype. Matrix components and their mechanical properties, such as stiffness, viscoelasticity, and viscoplasticity, influence diverse cellular processes, such as proliferation, migration/invasion, stemness, and cell differentiation. By interaction with ECM, cells sense mechanical cues and respond by triggering the activation of signaling cascades involved in cell survival, rearrangement of the cytoskeleton, activation of transcriptional regulators, and chromatin accessibility [157].

**Fig. 3:**
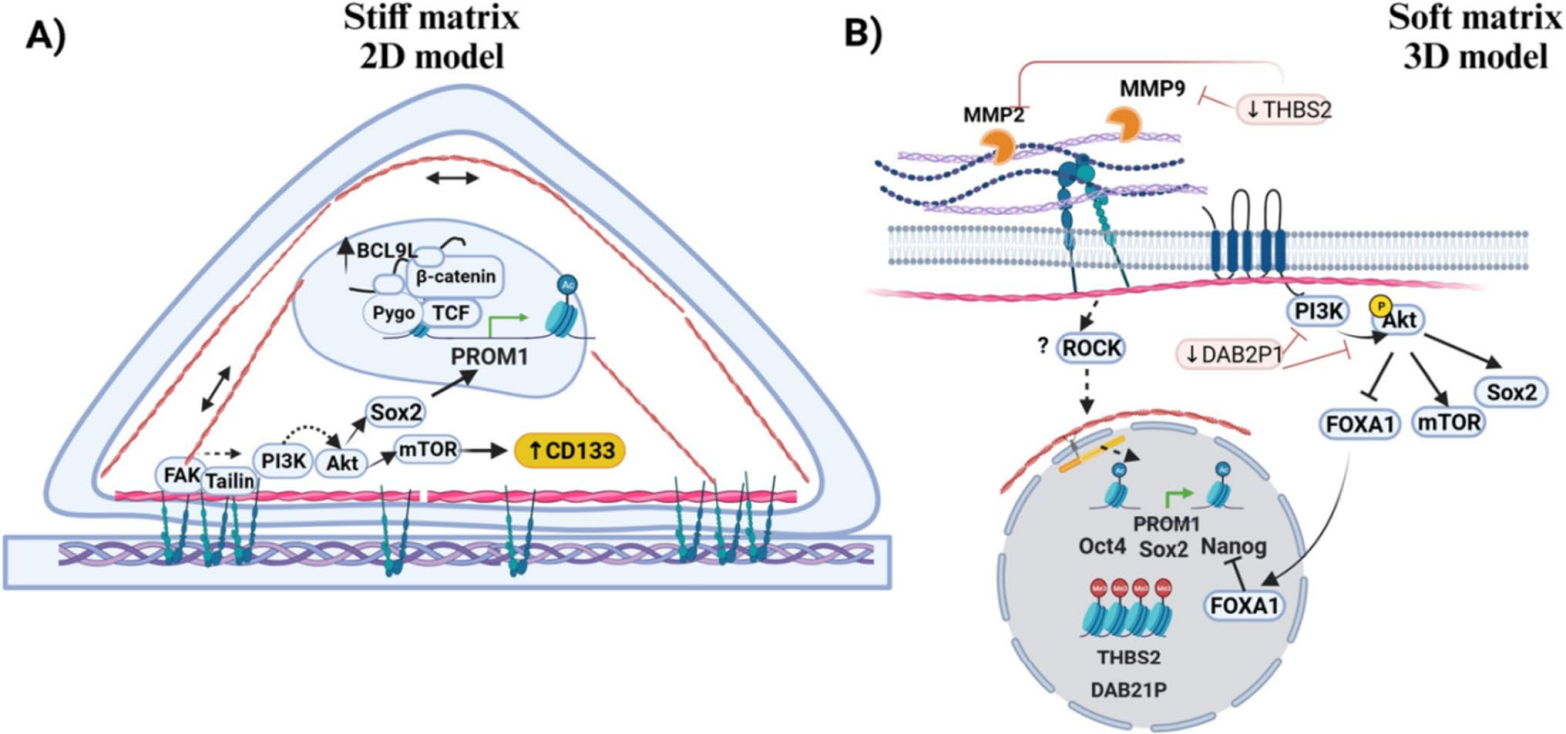
Mechanotransduction signaling mediated by integrin interaction with extracellular matrix (ECM) modulates CD133 expression in response to stiffness changes. The impact of ECM-cell interaction in CD133 levels can differ between traditional 2D (monolayer) and 3D in vitro models. **A** Monolayer cultures on stiffer matrices promote the establishment of focal adhesions that transduce mechanical forces in signaling activation, such as PI3K signaling. Also, contractile forces transmission to the nucleus can modulate pathways at the transcriptional level modulating transcription coactivators, transcription factors, and chromatin accessibility. By contrast, cells embedded in ECM sense mechanical clues such as stiffness via integrins, but the downstream signaling is unclear. **B** Soft matrices in 3D models increased CD133 expression through the improved expression of Sox2, Oct4, and regulation of chromatin accessibility to PROM1 promoter while repressing the expression of THBS2. CD133 in cell surface improves PI3K /Akt signaling, which can increase Sox2 and Nanog levels. Created with BioRender.com

Although there are limited studies about how ECM affects CD133 expression, ECM impact could depend on matrix composition, cell type, and cellular context. Also, mechanotransduction signaling can differ between traditional 2D (monolayer) and 3D in vitro models, and thus its impact on cellular processes could diverge (Fig. 3). For instance, You et al. (2016) found that matrix stiffness enhances CD133 expression in monolayer cultures of HCC cells through the integrin β1/Akt/mTOR axis [158]. Cells sense mechanical cues using integrin β1 that bind to Collagene-I fibers and trigger downstream signaling (Fig. 3A). By contrast, Ng et al. (2021) suggested that local soft spots in the HCC tumors maintain the CD133 + subpopulation in the 3D matrix gel system and xenograft models. Soft matrix through mechano-epigenetic regulation induces chromatin accessibility on CD133 promoter by activating histone modifications leading to enhanced expression **(**H3K4me3 and H3K9ac), while repressing THBS2 (thrombospondin 2) promoter through transcriptional silencing markers (H3K9m3 and H3K27m3**)** [159] (Fig. 3B). THBS2 has been considered an ECM-modifying enzyme that regulates extracellular matrix deposition leading to ECM stiffness. Therefore, CD133 + cells with THBS2 low expression promote a feedback loop that allows them to sustain the surrounding soft microenvironment, thereby facilitating invasion and metastasis by ECM remodeling.

Whereas several studies in 2D models of hepatocellular carcinoma have demonstrated a high correlation between increasing stiffness (that resembles liver fibrosis/cirrhosis) and hepatocellular carcinoma development [160–163], 3D in vitro systems with matrices that resemble physiological ECM (native tissue and tissue disturbed by disease) and studies of mechanical properties of ECM in vivo will allow unveiling the role of ECM-cell interactions that mediate disease evolution.

Without overlooking, 2D culture models have enabled to study the mechanotransduction pathways implicated in different ECM interactions and unveil the cellular behavior under the diverse stimuli. Integrin signaling is the central mechanism in 2D models by which cells sense ECM stiffness. While some studies reported the importance of integrins in mediating changes in CD133 levels [158, 164], others do not explore the upstream signals of transcriptional regulators [165, 166]. You et al. (2016) and Tao et al. (2021) reported CD133 upregulation by increasing ECM stiffness in HCC and glioblastoma, respectively. Akt/mTOR activation downstream of integrin-ECM interaction in HCC could increase CD133 synthesis via mTORC1 activation and PROM1 gene expression via Akt-mediated SOX2 protein stabilization [158, 165]. On the other hand, in glioma cell lines, increasing ECM stiffness causes an increase in BCL9L, a transcriptional coactivator of the β-catenin/TCF complex that favors the gene transcription of its target genes, including PROM1 (gene-coding CD133 protein). mTORC1 inhibition and β-catenin signaling inhibition override the CD133 increased levels triggered by the matrix in HCC and glioblastoma, respectively.

Alternatively, soft matrices can also induce changes in the CD133 expression in 3D models of HCC, as previously mentioned. Local soft spot microenvironment enhances CD133 expression via epigenetic modifications; nevertheless, the mechanism that regulates this process is unclear yet, and there are contrasting studies on changes in chromatin accessibility mediated by mechanical clues [157]. In addition, soft matrices in 3D models compared to rigid plastic plates enhance the CD133 mRNA levels and other cancer stem markers such as Oct4, Sox2, and Nanog in CRC cell lines. Although CD133 levels correlated with Nanog upregulation and low levels of DAB21P [166], a negative regulator of PI3K/Akt signaling, overexpression of DAB2P1 did not disturb CD133 mRNA levels, which suggest that other mechanisms triggered by matrix mechanical clues could be mediating this effect.

### Regulation by Tumor Associated Cells

Tumoral cells constantly are exposed to numerous secreted factors from tumor-associated cells that regulate cell behavior, quiescence, and stem/differentiation state. Numerous studies have demonstrated that CD133 expression increases as an outcome of the cell communication between tumoral cells and its niche in several cancers through cytokines secretion such as interleukins, TNFα/β, HGF, adipokines, and TGFβ (Table 1).

**Table1:** CD133 regulation in tumor cells by soluble factors in the microenvironment

Cancer	Tumor-associated cells^1^	Secreted factors	Signaling induced in tumor cells	Reference
HCC	Stellate cells (CAFs transformation)	IL6/HGF	ND	[167, 168]
		HGF	ND	[169]
		SCUBE1	Shh/GLI-1	[170]
	Ectopic	IL6	STAT3/NFkB	[171]
	Ectopic	LPS	NFkB/HIF-1α	[172]
HBx-infected hepatoma cells	HUEVECs	TGFβ	TGFβ signaling	[173]
Thyroid cancer	M2-like macrophages	Wnt ligands? (ND)	β-catenin	[174]
	Neurons	Acetylcholine	M3R/p-Src/CD133/Akt	[175]
Glioma	T Regs	TGF beta	NFKB/IL6/STAT	[176]
	Ectopic	Leptin	ObR/STAT/Notch	[177]
Medulloblastoma	Astrocytes	DN	DN	[178]
Colo-rectal cancer (CRC)	Ectopic	IL25	AMPK/SHH/GLI1	[179]
	Myofibroblasts	IL6, IL8	STAT3/Notch	[180]
	Jurkat cells	TNFβ	NFkB	[181]
	MSCs (placenta)	IL8	CXCR2/ERK	[182]
	CD206 + macrophages	Cytokines	ND	[183]
	Tumor associated macrophages	TAM-conditioned medium	STAT3	[184]
Ovarian cancer	Ectopic/CD68 + macrophages	IL17	NFkB	[185]
	M2-like macrophages	IL8	ND	[186]
Oral Cancer	M1-like macrophages	TNFα, IL6, IL1β	ND	[187]
	CAFs	CXCL2/IL6	ND	[188]
Pancreatic cancer	Stellate cells (CAFs transformation)	IL6	STAT3	[189]
	Adypocites	Conditioned medium	ND	[190]
Lung cancer	BM-MSC	IL6	IL6R/STAT	[191]
Esophageal squamous carcinoma	Ectopic	IL23	STAT3	[192]

^1^Soluble factors secreted by tumor-associated cells are referenced; in various studies its effect was evaluated by ectopic expression to resemble tumor-associated cells secretion. ND: no detected or not evaluated

Interleukins appear as the most studied secreted factors involved in CD133 upregulation by macrophages, cancer-associated fibroblasts (CAFs), mesenchymal stem cells (MSCs), and adipocytes in local niches. Interleukins can induce STAT3 activation and its target genes expression in tumoral cells [180, 189, 191, 192]. STAT3 binds to the PROM1 promoter and increases CD133 protein levels [171]. But also, STAT3 cross-talking with other signaling pathways produces a robust response to external signals to improve CD133 expression and cancer stem features. STAT3 can activate Notch signaling through improved Notch ligands and receptors expression. Kim et al. (2021) reported that IL6/IL8 secreted by myofibroblasts in CRC microenvironment increased CD133 + /CD44 + cell subpopulation [180] probably through STAT3/Jagged1 axis that triggers Notch signaling activation in tumor-adjacent cells [193] which has shown to regulate positively CD133 expression [194, 195].

High-fat diets and an imbalance between energy expenditure and food intake promote fat accumulation leading to adipocyte tissue dysfunction, metabolic disorders, and chronic inflammation associated with cancer development. Leptin is an adipokine secreted predominantly by adipocytes to regulate food intake and metabolism. Its levels are highly upregulated in obese individuals and linked to the development of several cancers because of its role in cell proliferation, angiogenesis, ECM-remodeling, pro-inflammatory niche maintenance, apoptosis resistance, and cancer stemness [196]. Leptin/STAT3/Notch axis enhances CD133 levels, clonogenic and self-renewal capacity, whereas STAT3 inhibitor, γ-gamma secretase inhibitor, and leptin antagonist (LDFI), abrogated leptin effect on stem cells functions in glioblastoma [177]. CD133 + cells tended to accumulate around adipocytes in pancreatic tumors. Kesh et al. (2022) reported that conditioned media from patient-derived adipocytes increased CD133, Sox2, and Oct4 expression, which suggests that peritumoral adipocytes are part of the homing niche that maintains CD133 + population partially because of interleukins secretion such as IL6 [190].

In addition, high-fat diets are associated with abnormal oxidative stress and hyperlipidemia. ROS overproduction produce higher levels of oxidized low-density lipoproteins (ox-LDL) that promotes tumor-associated macrophage (TAM) activation in cancer. Interleukins produced by CD206 + macrophage cells could induce STAT signaling in tumoral cells, which upregulate CD133 and CD44 expression [183]. Further, ox-LDL could trigger ROS/NFkβ/interleukins that act in an autocrine manner to activate STAT3 and subsequent target gene expression [197]. Also, other soluble factors such as TGFβ and TNFα/β from T Regs and T cells respectively, activate NFkB/Interleukins axis to probably reinforce STAT signaling in a feedback loop [176, 181]. IL17 promotes CD133 upregulation and enhanced clonogenic capacity, partially via NFkB activation in ovarian cancer cells. IL17 secretion by CD4 + lymphocytes and CD68 + macrophages in the local niche close to CD133 + ovarian cancer cells favor CD133 + cell maintenance [185]. IL17B could enhance CD133 expression via the Akt/β-catenin axis in other cancers [198].

Beyond STAT signaling and its crosstalking with other signaling pathways, stromal cells can induce Hedgehog, and Wnt signaling activation led to CD133 upregulation in tumoral cells. Endothelial cells and CAFs promote CD133 expression through Hedgehog pathway activation through Shh and SCUBE1 secretion in glioblastoma and HCC, respectively [170, 199]. SCUBE1 is a glycoprotein involved in Shh ligand secretion and membrane release; its overexpression in CAFs raises Shh, SMO, and GLI-1 levels with a concomitant increase in CD133 levels, whereas its downregulation displays an opposite effect, resulting in reduced migration and sphere-forming ability in tumoral cells [170]. Also, IL25 in CRC tissues could positively regulate Hedgehog signaling by p-AMPK reduction leading to Gli-1 stabilization and nuclear translocation in cancer cells. However, it is not clear how IL25 disturbed AMPK levels and its phosphorylation in CRC [179]. M2-like macrophages could secrete Wnt ligands that enhance CD133, Oct4, and cMyc expression through β-catenin stabilization and nuclear translocation [200]. M2-like macrophages improved clonogenic anchorage-independent capacity, cell migration, and invasion in thyroid cancer cells, whereas Wnt/β-catenin signaling inhibition abolished it [174].

**Fig. 4:**
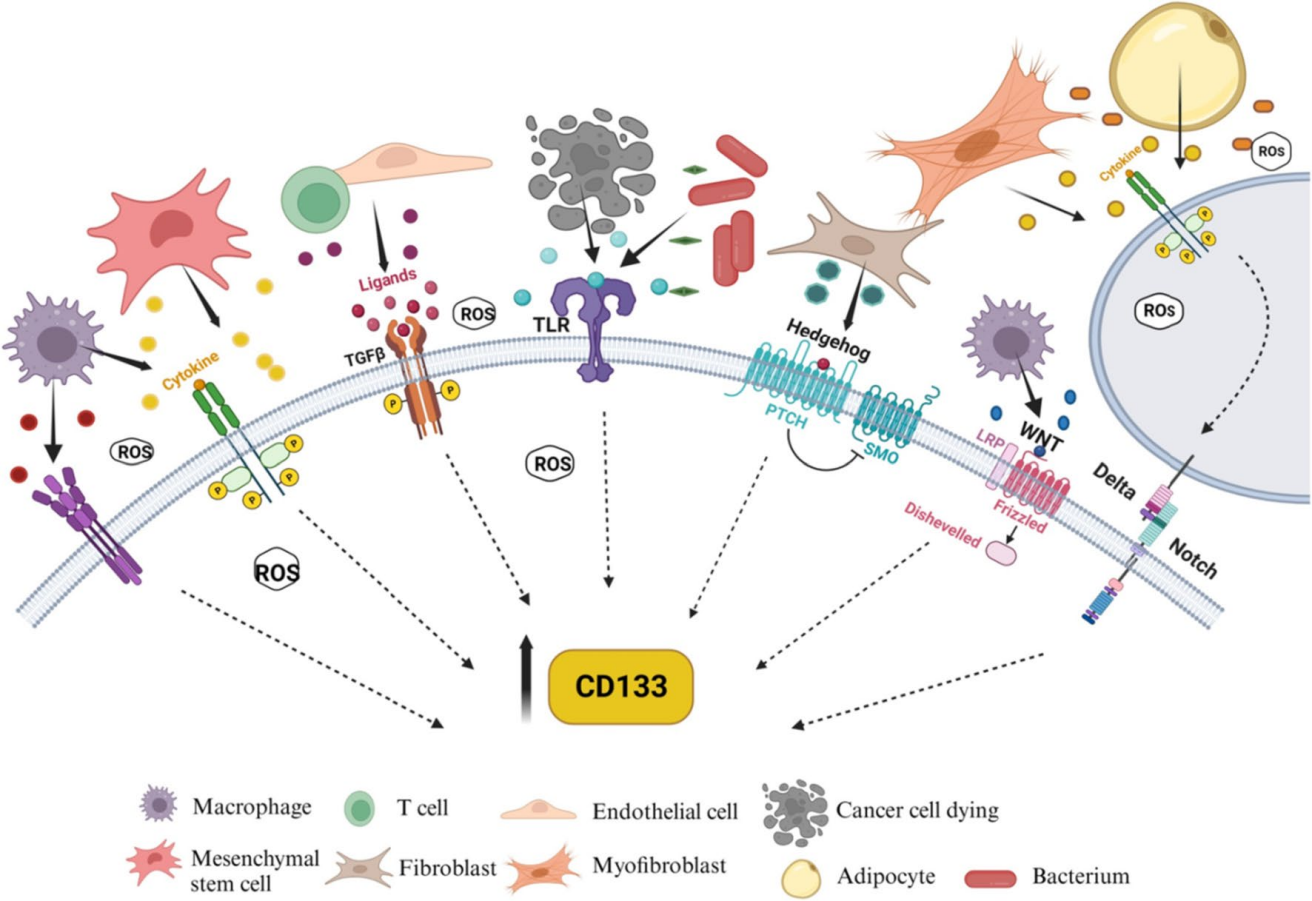
The tumor microenvironment regulates CD133. Tumor-associated cells modulate CD133 levels in cancer cells through the secretion of cytokines, growth factors, HMGB1, Shh, and Wnt ligands, and so forth. Also, toxins secreted such as LPS by bacteria could modulate CD133 expression [172, 184, 207].These factors induce signaling pathways that positively regulate CD133 expression. Created with BioRender.com

Endothelial cells also contribute to CD133 + subpopulation maintenance in perivascular niches through TGFβ secretion and downstream signaling activation in hepatoma cells infected with the hepatitis B virus. CD133 appears essential to mediate the TGFβ effect on the EMT process in HBx-infected hepatoma cells [173]. TGFβ signaling regulate CD133 expression in a dose-dependent manner in part through the inhibition of DNA methyltransferases DNMT1 and DNMT3β expression, resulting in CD133 (PROM1) promoter P1 methylation reduction, which favors its activation [51]. On the contrary, CD113 + cells of MHCC97H (HCC cell line) exhibit DNMT1 higher expression than CD133- cells; its knockdown results in stemness loss, miR34a transcription, and subsequent FOXM1 downregulation. DNMT1/miR3a/FOXM1 axis plays an essential role in cancer stem-like traits in some liver cancer [201]. FOXM1 regulation seems crucial in cancer progression and enhanced stemness in cancer [202–204]. DNMT1 loss could be detrimental to tumoral cells because of the multiple targets that it modulates (Fig. 4).

On the other hand, beyond CD133 levels regulation by tumor-associated cells, CD133 activity can be also modulated by secreted factors. Wang et al. (2020) found that acetylcholine secreted by neurons associated with thyroid cancer cells induces Y828 phosphorylation of CD133 and downstream PI3K/Akt activation, which enhances self-renewal and restrains CD8 + T cell cytotoxicity [175]. Acetylcholine binding to the M3R receptor and subsequent Src activation was essential to mediate CD133 function. Also, Wei et al. (2019) reported that CD133 + cells interact preferentially with lymphatic endothelial cells in HCC tissues, and its interaction promotes IL17 secretion by endothelial cells that stimulate STAT3 signaling and subsequent PD-L1 upregulation in tumoral cells. This interaction seems to depend on high-mannose glycans expressed on CD133 + cells and mannose receptors on endothelial cells; inhibition of this physical interaction abolished IL17 secretion and CD133 + cells' resistance to CD8 + T cells [205]. This evidence suggests that hindering the CD133 function in tumor cells could impair not only self-renewal and tumorigenicity, but also the ability of CD133 + cells to modify their niche. LDN193189 small molecule interacts with the C-terminus of CD133 and hampers p85 recruitment and downstream signaling, impairing self-renewal and tumorigenicity of CD133 + cells in HCC. Also, LDN193189 downregulates galectin-3 transcription by impairing Y828/Y852 phosphorylation of CD133 in HCC cells, leading to CD8 + T cells activation [206].

### Cytotoxic Stress (Chemotherapy and Radiotherapy)

The acquisition of CD133 or pre-existence of CD133 + cells in the tumors is frequently associated with cancer stem-like cells with enhanced resistance to conventional therapy. CD133 promotes chemoresistance partially through Akt activation, which can modulate the activation of several proteins involved in drug efflux, apoptosis, and cell survival. CD133/Akt inhibits pro-apoptotic proteins and promotes antiapoptotic proteins and MDR1 expression via NFKB [208–210]. Also, CD133/Akt inhibits JNK and p38 downstream signaling involved in drug-induced apoptosis and promotes p53 degradation through MDM2 stabilization [211]. Whereas P53 could restrict CD133 expression under normal and stress conditions, CD133 upregulation abolishes p53 tumor suppressive function, indicating a mutual negative regulation that depends partially on the mutational background.

CD133 loss in different cancers can sensitize resistant cells to conventional chemotherapy by inducing PI3K/Akt signaling reduction [212–214]. In addition, CD133 depletion frequently is accompanied by the suppressed expression of the pluripotent stem genes and slow proliferation phenotype in cancer [4, 215, 216], which suggests that CD133 downregulation could impact several cellular processes via dependent and independent of PI3K/Akt signaling in a context-dependent manner. CD133 acquisition during therapy emerges in response to cytotoxic stress as an adaptation mechanism to promote survival. Hereafter we will focus on the possible mechanisms triggered in the tumoral cell by its surrounding niche that promotes CD133 expression during or after drug treatment.

Tissue damage, inflammatory response, and cell death caused by chemotherapy or radiotherapy, can induce pathways activation or inhibition that promotes cell survival and tumor progression. Hou et al. (2020) reported that irradiated mesenchymal cells (IR-MSCs) increase CD133 expression and tumor growth in HCC through autocrine Wnt3a/β-catenin signaling activation. Disrupting CBP-β-catenin interaction abolishes the IR-MSCs effect, but how IR-MSCs stimulate Wnt ligand secretion in tumoral cells is still unclear [217]. Cytokines secreted by stromal or cancer cells contribute to chemotherapy differential outcomes. Colony-stimulating factor (GM-CSF) upregulation and secretion after 5FU and cisplatin treatment trigger STAT3 activation in residual gastric cancer cells. GM-CSF increased miR-877-3p expression, which suppresses SOCS2 (suppressor of cytokine signaling 2), a negative JAK/STAT3 signaling regulator, driving CD133 + cells to expansion and Sox2, Oct4, KLF4, and h-TERT upregulation [218]. IL6 upregulation induced by MUC1/EGFR axis in tumoral cells results in CD133 + subpopulation expansion after chronic and acute paclitaxel treatment. MUC1 increased expression after paclitaxel treatment supports acquired chemoresistance via MUC1-C/EGFR complex nuclear activity, thereby EGFR inhibition by Erlonitib sensitized cells to paclitaxel, abrogated CD133 + cells enrichment and prevented disease relapse of cervical cancer [219].

HCC early recurrence after radio-frequency ablation (RFA) treatment was associated with CD133 + cell growth partially due to VEGF and CXCL10 elevated levels after the treatment [220, 221]. Microvascular and tissue damage after RFA treatment could trigger HIF1α stabilization and subsequent VEGF expression to promote local angiogenesis and residual cell expansion characterized by CD133 enhanced expression [222]. Nanog increased expression induced by VEGF raises the frequency of CD133 + cells and enhances its clonogenic capacity [221]. Disturbing VEGF-VEGFR2 interaction abrogates CD133 expansion driven by VEGF. Blocking CXCL10-CXCR3 interaction, CXCL10 fails to increase the c-Myc levels and proportion of CD133 + cells [220], but the molecular mechanism downstream VEGFR and CXCR3 after RFA treatment is unclear. In addition, Adini et al. (2013) demonstrated that CD133 interacts with VEGF, promoting its stabilization and dimerization, which favors VEGF binding to its receptor in endothelial cells and tumoral cells, indicating a possible feedback wherein CD133 potentiates VEGF pro-angiogenic function in endothelial cells, and survival in tumoral cells via VEGF/Bcl-2 axis [223].

Tumors undergoing chemotherapy or radiotherapy display HMGB1 increased levels in the extracellular space that favors the acquisition of cancer stem-like features. HMGB1, a non-histone nuclear DNA binding protein involved in chromatin remodeling and gene transcription, also acts as an inflammation mediator by binding to TLR receptors. HMG1B can be released by damaged, dying cells, stromal cells or actively secreted by cancer cells in response to an external stimulus such as hypoxia and cytokines [224, 225]. Zhang et al. (2019) demonstrated that CD133-negative tumoral cells acquired CD133 expression after exposure to HMGB1-released by irradiated-tumoral cells. HMG1B binds to TLR2 and induces YAP stabilization and YAP/HIF-1α nuclear association, leading to pluripotency gene transcription. YAP binding to pluripotent gene promoters was dependent on HIF1α upon HMGB1 stimulus. Deficiency of TLR2 and HIF-1α, abolished CD133 expression and clonogenic ability induced by HMG1B. TLR2/YAP/HIF1α inhibitors delayed pancreatic tumor relapse in mice undergoing X-ray irradiation treatment [226]. Also, in response to temozolomide, GBM cells secrete HMG1B that triggers lncRNA NEAT1 (nuclear paraspeckle assembly transcript 1) upregulation favoring β-catenin/TCF signaling [227]. NEAT1 could regulate positively or negatively the Wnt signaling through mRNA or protein stabilization of proteins involved in the signaling cascade and the transcriptional complex [228–230].

## Conclusions and Perspectives for Therapeutic Strategies

The accuracy of CD133 as a biomarker for cancer stem-like cells remains controversial, primarily due to the presence of CD133-negative cells that also possess a tumorigenic potential and enhanced chemoresistance in several tumors [83, 231, 232]; and plasticity of stemness markers expression [233]. These findings suggest the existence of various subpopulations with distinct surface marker expressions with potential for tumor initiation and probably with a differential contribution to cancer progression, such as proliferation, latency, migration, and invasiveness. Additionally, the tumor microenvironment influences CD133 expression in tumoral cells and its modulation could be part of an adaptive response.

Remarkably, CD133 has been regarded as a therapeutic target for eradicating drug-tolerant cancer cells and preventing cancer recurrence in various cancer types. Targeting CD133 enhances cell sensitivity to conventional chemotherapy by suppressing the overexpression of CD133 or preventing the enrichment of CD133 + cells in response to cytotoxic stress. In gastric cancer, the combined treatment of cisplatin and anti-CD133 CAR-T cells has demonstrated significant antineoplastic activity compared to individual therapeutic strategies [234].

Targeted immunotherapy utilizing antibodies that engage NK cells and T cells to CD133 + subpopulation has shown reduced outgrowth of CD133-overexpressing glioblastoma (GBM) tumors and decreased tumor burden in mice with myeloid leukemia, without impairing hematopoietic stem cells, at concentrations effective against tumor cells [235, 236]. Furthermore, the development of vaccines incorporating modified CD133 mRNA into dendritic cells (DCs) has induced cytotoxic activity of both CD4 + and CD8 + T cells against triple-negative breast cancer (TNBC) and GBM cells, resulting in reduced tumor growth and increased survival in vaccinated mice [237, 238]. Other drug delivery systems that employ CD133 antibodies and CD133 peptides to eliminate CD133 + cells have shown heterogeneous efficacy and discrete responses in different in vitro and in *vivo* cancer models [239–241], likely due to the initial antigen expression on the cell surface and changes in its expression during treatment [242].

As these therapeutic strategies rely on CD133 extracellular domain recognition on the cell surface, understanding its internalization and potential functions in other cellular compartments may enable the development of more reliable CD133-targeted therapies with fewer limitations. Since CD133 expression is not limited to cancer stem-like cells and also stem/progenitor cells and mature cells of various tissues, such as bone marrow, brain, kidney, liver, pancreas, and retina exhibit CD133, it is of paramount importance to elucidate the structural differences caused by post-translational modifications and alternative splicing variants that may mediate diverging functions between tumor and non-tumor cells, which is essential for designing precise therapeutic strategies against cancer and particularly CD133 + subpopulations. Additionally, the potential compensation by prominin-2 should be considered when developing therapies for particular cancer types.

## Data Availability

Data sharing not applicable to this article as no datasets were generated or analyzed during the current study. Other materials such as Figures are within the article.
